# Proteomic Analysis of Fetal Ovaries Reveals That Primordial Follicle Formation and Transition Are Differentially Regulated

**DOI:** 10.1155/2017/6972030

**Published:** 2017-02-07

**Authors:** Mengmeng Xu, Long Che, Zhenguo Yang, Pan Zhang, Jiankai Shi, Jian Li, Yan Lin, Zhengfeng Fang, Lianqiang Che, Bin Feng, De Wu, Shengyu Xu

**Affiliations:** ^1^Key Laboratory of Animal Disease-Resistance Nutrition and Feed Science, Ministry of Agriculture, Sichuan Agricultural University, 211 Huimin Road, Wenjiang District, Chengdu, Sichuan 611130, China; ^2^Key Laboratory of Animal Disease-Resistance Nutrition, Ministry of Education, Wenjiang, China

## Abstract

Primordial follicle formation represents a critical phase of the initiation of embryonic reproductive organ development, while the primordial follicle transition into primary follicle determines whether oestrus or ovulation will occur in female animals. To identify molecular mechanism of new proteins which are involved in ovarian development, we employed 2D-DIGE to compare the protein expression profiles of primordial follicles and primary follicles of fetal ovaries in pigs. Fetal ovaries were collected at distinct time-points of the gestation cycle (g55 and g90). The identified proteins at the g55 time-point are mainly involved in the development of anatomical structures [reticulocalbin-1 (RCN1), reticulocalbin-3 (RCN3)], cell differentiation (actin), and stress response [heterogeneous nuclear ribonucleoprotein K (HNRNPK)]. Meanwhile, at the g90 stage, the isolated proteins with altered expression levels were mainly associated with cell proliferation [major vault protein (MVP)] and stress response [heat shock-related 70 kDa protein 2 (HSPA2)]. In conclusion, our work revealed that primordial follicle formation is regulated by RCN1, RCN3, actin, and HNRNPK, while the primordial follicle transformation to primary follicle is regulated by MVP and HSPA2. Therefore, our results provide further information for the prospective understanding of the molecular mechanism(s) involved in the regulation of the ovarian follicle development.

## 1. Introduction

Proper gilt development is critical for yielding productive pigs and consequently is essential for sustaining economic efficiency. Conditioning of the ovary can be adjusted to maximize the reproductive lifetime of gilts. Generally, studies on ovary development are focused on primordial follicle formation and transition into primary follicle. Gilt fecundity is determined at the time when the fetal primordial follicle pool is established, while any abnormalities in the formation of primordial follicles can result in infertility [1]. The primary follicles develop from a reserve of primordial follicles generated early in life [2]. Although the total number of primordial follicles is not altered in the offspring, the number of activated cells can determine the lifetime of reproduction [2]. Ovarian dysplasia is a disorder characterized by abnormal formation or differentiation of primordial follicles which can lead to failure or early estrus, causing a reduced lifetime reproduction.

In the fetal ovaries of pigs, the germ cells migrate in clusters to the gonad [3] and begin to enter meiosis at day 47 of gestation [4]. The cells in these clusters undergo apoptosis while the cluster cysts are broken down to enclose the immature oocytes and form the primordial follicles [5]. Eventually, each primordial follicle consists of one immature oocyte which is surrounded by several somatic granulosa cells. Through a series of activation steps the primordial follicular cells are transformed into primary follicles [5] and these processes are critical for ovarian development [6]. The process of primordial follicle formation in pigs begins at day 56 of gestation and transformation into primary follicle is first observed at day 90 of gestation (g90) [5].

The heat shock protein (Hsp) is mainly responsible for maintaining appropriate internal environment in the developing ovary. The extensive studies have revealed that an increase in Hsp synthesis may incite oxidative stress that is connected to the risk of reproductive diseases and damage to the ovaries [7]. In addition to Hsp, several other genes have been identified to be involved in the development of the ovary [8–10]. However, the regulatory mechanism(s) leading to ovary and follicle development and the molecular interactions of many genes/proteins involved in these pathways need to be investigated further.

2D-DIGE technology is a widely used method which allows the direct comparison of samples with distinct proteomic profiles in order to identify differentially expressed proteins. Moreover, investigation of the fetal ovary proteome in pigs can be crucial for detecting key physiological and biological changes which regulate follicular development. Therefore, the objective of the present study was to compare two distinct follicular developmental stages [at day 55 (g55) and day 90 (g90) of gestation] for detecting differentially abundant proteins and identifying potential markers for primordial follicle formation or transition to primary follicle. Our results offer a new insight towards an understanding of the molecular basis of follicle formation and differentiation.

## 2. Materials and Methods

### 2.1. Gilts and Tissue Sample Collection

All the experimental procedures performed in this study were approved by the Guide for the Care and Use of Laboratory Animals prepared by the Animal Care and Use Committee of Sichuan Agricultural University. To obtain pregnant animals, similar weight (135.54 ± 0.66 kg) female Yorkshire gilts (*n* = 8), at the 3rd estrus, were mated twice by artificially insemination with the same Yorkshire boar. The diet of the animals was formulated to meet nutrient requirements as recommended by the National Research Council 2012 (NRC 2012), which includes 13.9% of crude protein, 0.69% Lys, 0.96% calcium, and 0.79% phosphorus. After artificial insemination, the pregnant gilts were housed individually and fed 2 kg/day (g0–g30) and 2.4 kg/day (g31–g90). All gilts were given ad libitum access to water. Four gilts were selected randomly at g55 to be anaesthetized with an intravenous injection of Zoletil 50 (0.1 mg/kg body weight; Zoletil 50 Vet, Virbac, Carros Cedex, France) and were slaughtered to obtain female fetal ovaries. The remaining 4 pregnant gilts were similarity slaughtered at g90.

### 2.2. Sample Collection

While under anesthesia, both ovaries were collected from all the female fetuses of the pregnant pig. One ovary was fixed in 4% paraformaldehyde solution, and the other was rapidly frozen in liquid nitrogen and stored at −80°C until further analysis.

Fixed fetal ovaries in 4% paraformaldehyde were routinely stained with hematoxylin and eosin. Briefly, the samples were dehydrated and embedded in paraffin and then were cut into 4 *μ*m slices on microtome. The stain of the slices was removed by washing with dimethylbenzene and dewaxing was followed by immersing the slice in different concentrations of ethanol. Finally, the samples were stained with hematoxylin, hydrochloric acid, and eosin, and the slices were imaged under bright field microscopy.

### 2.3. Protein Sample Preparation

Overall, twelve fetal ovary tissue samples were collected randomly from 4 pregnant gilts, at g55 or g90 stage, for proteomic analysis. The 3 randomly selected fetal ovaries from each of the pregnant gilts were pooled in one sample for further testing. The samples were smashed using a pestle in 2 M thiourea (Guangzhou Chemical Reagent Factory, Guangzhou, China), 7 M urea (Guangzhou Chemical Reagent Factory, Guangzhou, China), phenylmethanesulfonyl fluoride (PMSF, Sangon Biotech (Shanghai) Co., Ltd., Shanghai, China), and 4% w/v CHAPS (Sangon Biotech (Shanghai) Co., Ltd., Shanghai, China). Following centrifugation at 13,000 ×g for 20 min at 4°C, acetone (Fisher, Saint Louis, USA) was added to the supernatant and the mixture was centrifuged at 13,000 ×g for 20 min, at 4°C. The precipitated proteins were then separated according to their mass and isoelectric point by two-dimensional difference in-gel electrophoresis (2D-DIGE). The concentration of the protein was determined using a 2D Quant kit (Amresco, LLC, OH, USA).

### 2.4. Protein Sample Labelling and Two-Dimensional Difference Gel Electrophoresis

These procedures were repeated twice. The internal standard sample of each gel was generated by pooling equal amounts of proteins from the synchronized samples (at g55 and at g90). Protein extracts from the standard and the fetal ovary samples at g55 and at g90 stages were labelled with Cy2, Cy3, and Cy5, respectively. The dyes were mixed in a final volume of 460 *μ*L with 1% dithiothreitol (Guangzhou Chemical Reagent Factory, Guangzhou, China), 1% IPG (immobilized pH gradient) buffer (GE Healthcare Life Sciences, NJ, USA), rehydration buffer (bromophenol blue) (Guangzhou Chemical Reagent Factory, Guangzhou, China) in 7 M urea, 2 M thiourea, and 4% PMSF. The mixture was loaded onto a 24 cm, pH 4–7 IPG dry strip for the first-dimensional isoelectric focusing after 12 hours of rehydration. The IPG strips were equilibrated in 8 mL equilibration solution containing 30% glycerol, 0.002% bromophenol blue, 6 M urea, and 4% SDS and subsequently mixed with 100 mM dithiothreitol for 15 min. Afterwards, the strip was cut away and iodoacetamide was added to obtain a final concentration of 250 mM (Guangzhou Chemical Reagent Factory, Guangzhou, China) in the same solution and a further incubation for 15 min was followed. The electrophoresis was performed according to a previously described method [11], with slight modifications. Briefly, for the first-dimensional electrophoresis, the following program was implemented: 300 V for 30 min, 700 V for 30 min, 1500 V for 1.5 h, 9000 V for 3 h, and, finally, 9000 V for 4 h; the electrophoresis was set at 52 KVh at all times. For the second dimension, the strips were transferred to a polyacrylamide gel for sodium dodecyl sulphate-polyacrylamide gel electrophoresis (SDS-PAGE). The proteins were separated by the following settings: 2 W/gel for 45 min and then at 17 W/gel for 4.5 h, until the bromophenol blue reached the bottom of the gels.

### 2.5. Gel Image Acquisition and Analysis

The Typhoon 9400 imager (GE Healthcare Life Sciences, NJ, USA) was used to scan for protein spots at wavelengths of 488/550 nm (cy2), 532/550 nm (cy3), and 633/550 nm (cy5), respectively. The 2D DIGE gel images were analyzed by the Image Master 2D platinum 7.0 software (GE Healthcare Life Sciences, NJ, USA) and the protein abundance changes for spot picking detection were calculated using cy3/cy2 and cy5/cy2 differential in-gel analysis ratios. Only spots with significantly different changes (*P* < 0.05) and in-gel ratios greater than 1.6 were excised from the silver stained gels for further analysis to identify specific proteins of interest.

### 2.6. Spot Picking and Enzymatic Digestion

Selected spots were automatically cut form gels, washed twice with Milli-Q water for 30 min, and destained in 50% methyl alcohol (Guangzhou Chemical Reagent Factory, Guangzhou, China). The spots were dissolved in 100 mM NH_4_HCO_3_ (Sigma-Aldrich Co. LLC, MO, USA) and the proteins were extracted with 50% acetonitrile (ACN; Sigma-Aldrich Co. LLC., MO, USA). The proteins were then digested with 1 *μ*g/*μ*L robust trypsin (Promega Corporation, WI, USA) for 30 min. Subsequently, coverage solution (10% ACN, Milli-Q water, and 50 mM NH_4_HCO_3_) was added, and the samples were incubated further for 16 h. Following digestion, the generated peptides were extracted with a solvent that consisted of 90% ACN and 2.5% trifluoroacetic acid (TFA; Promega Corporation, WI, USA) for 30 min. Finally, the peptides were vacuum-dried.

### 2.7. Matrix-Assisted Laser Desorption Ionization Time-of-Flight Mass Spectrometry (MALDI-TOF-TOF MS) Analysis

After vacuum drying, the dried peptides were resuspended in 30% ACN, 0.1% trifluoroacetic acid (TFA), and Milli-Q water in a final volume of 1.5 *μ*L. Subsequently, 0.8 *μ*L of the dissolved peptides was mixed with 0.5 *μ*L of 5 mg/mL *α*-cyano-4-hydroxycinnamic acid (50% ACN containing 0.1% TFA) and the mixture was loaded onto a metal sample plate which was then air-dried at room temperature. Finally, the MALDI-TOF-TOF (Bruker Daltonik GmbH, Bremen, Germany) was used to perform MS analysis. The UA laser conditions were set at 355 nm wavelength, 200 Hz repetition rate, and 30 kV accelerate voltage. A Flex Analysis (Bruker Daltonik GmbH, Bremen, Germany) was used to filter the background noise generated from baseline peaks and to detect genuine signal peaks. In order to identify the proteins which produced the corresponding signal peaks, we compared our data to the NCBI database (https://www.ncbi.nlm.nih.gov/) BioTools (Bruker Daltonik GmbH, Bremen, Germany). For this analysis we have utilized a unique identification code, the values of sequence coverage, cleavage of trypsin, oxidation of variable modifications, the relative molecular mass, and the PI value. The entire query builder was run according to the following settings: 800–4000 Da molecular range of peptides, apparent pI, and apparent Mr error range: unlimited, max missed cleavage 1, peptide tolerance of 50 ppm, and fragment ion mass tolerance of 0.6 Da. In this study, we have selected proteins with features that showed a significant match (*P* < 0.05) and a score higher than 89. Finally, we also identified and analyzed proteins within the KEGG (Kyoto Encyclopedia of Genes and Genomes) metabolic pathway (http://www.kegg.jp/).

## 3. Results

### 3.1. Histological Characterization of Fetal Ovaries

**Figure 1:**
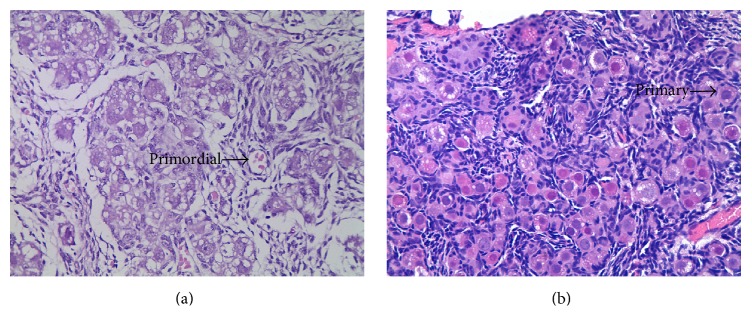
Photomicrograph of ovarian tissue from fetal ovary at day 55 and day 90 of gestation with hematoxylin and eosin staining. (a) At day 55 of gestation of fetal ovary; (b) at day 90 of gestation of fetal ovary; primordial: primordial follicle; primary: primary follicle. Both (a) and (b) are with original magnification: 400x.

In order to investigate whether our sample collection method correctly represented the differential follicle stages, we imaged the fixed ovaries and the follicles were classified as primordial or primary accordingly. A primordial follicle was defined by the presence of an individual oocyte surrounded by a single layer of flattened follicular cells, while for the primary follicles the classification was based on the presence of an oocyte surrounded by a single layer of cuboidal follicular cells. As shown in Figure 1, we have confirmed the presence of the primordial follicles at the g55 stage and the primary follicle of the fetal ovary at stage g90. Therefore, the samples collected from the corresponding animals were suitable for comparison of the two differential stages and were used for further analysis.

### 3.2. 2D-DIGE Analysis and Identification of Differentially Abundant Proteins

**Figure 2:**
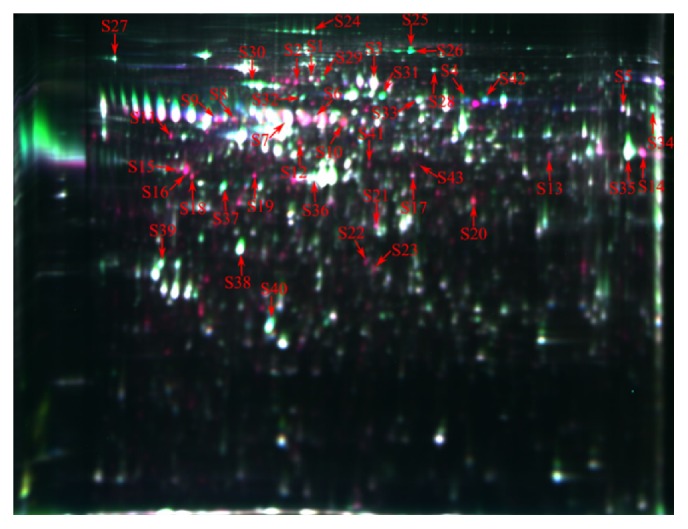
Representative 2D-DIGE gel image of differentially expressed proteins of fetal ovaries at day 55 and day 90 of gestation. The proteins extracted from the fetal ovaries at day 55 and day 90 of gestation samples were labelled with cy3 and cy5, respectively. An internal standard protein sample (a mixture of fetal ovaries at day 55 and day 90 of gestation samples) was labelled with the Cy2 dye. The number in the figure corresponds to the number shown in Table 1.

Approximately 1397 protein spots were detected, out of which 50 exhibited significant changes (ratio_g55/g90_ > 1.5 or ratio_g90/g55_ > 1.5, *P* < 0.05) between the two distinct ovarian developmental stages of the Yorkshire pig fetuses. Forty-three identified proteins (ratio_g55/g90_ > 1.6 or ratio_g90/g55_ > 1.6, *P* < 0.05) were selected for MALDI TOF-TOF MS analysis (Figure 2). Our results showed that Yorkshire fetal ovaries at g55 stage of gestation had 25 upregulated and 18 downregulated proteins compared with the g90 time-point (Table 1). These proteins were highly related to anatomical structure development, cytoskeleton organization, cell differentiation, cell proliferation, and response to stress. 39.5% of the differentially expressed proteins were involved in structural organization (20.9%, 9 proteins) and cell differentiation and/or proliferation (18.6%, 8 proteins), while 11.6% related to stress responses. Specifically, the proteomic analysis results indicated that the protein levels of the reticulocalbin-3 (RCN3), reticulocalbin-1 (RCN1), actin, and the heterogeneous nuclear ribonucleoprotein K (HNRNPK) at g55 stage were higher than those at g90. In contrast, the protein content of the major vault protein (MVP) and the heat shock-related 70 kDa protein 2 (HSPA2) at g55 were lower than at the g90 time-point.

### 3.3. Functional Categories Analysis

**Figure 3:**
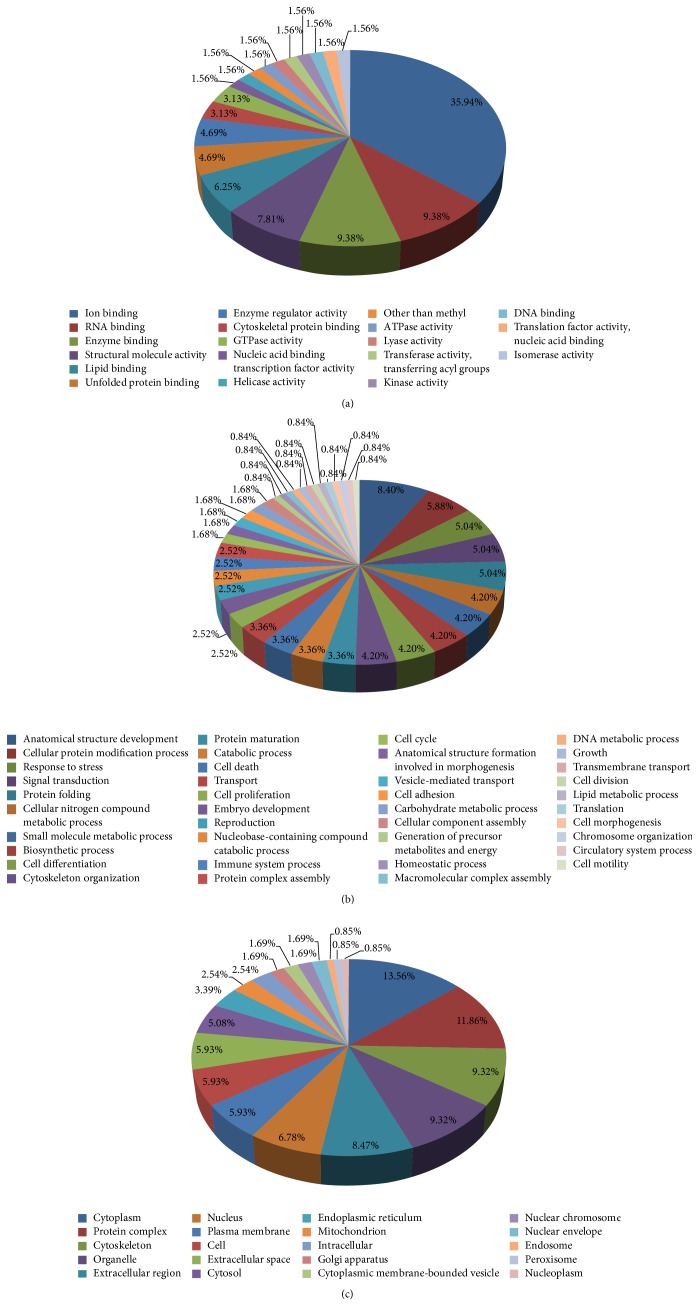
Functional annotations of all identified proteins. All identified proteins were functionally annotated in GO database according to their molecular function (a), biological process (b), and cellular component (c).

To reveal the biological function of the identified proteins of the fetal ovaries we performed a Gene Ontology (GO) annotation search using the NCBI (https://www.ncbi.nlm.nih.gov/) and porcine databases (https://www.ncbi.nlm.nih.gov/protein/?term=txid9822[Organism:exp]). GO annotations were available only for the 37 identified proteins; 30 proteins were grouped according to their molecular function, 28 according to biological processes, and 28 proteins according to cellular components annotations (see Table S1 of the Supplementary Material available online at https://doi.org/10.1155/2017/6972030). The assignment of the identified proteins to a particular group is presented in Figure 3. Based on cellular component GO analysis, 13.56% of the differentially expressed proteins were concentrated in the cytoplasm, 11.86% in protein complexes, 9.32% in the cytoskeleton, and 6.78% in the nucleus. Interestingly, our results revealed that most of the proteins located in the nucleus were related to major stress response processes (Table S1). Finally, the most common molecular functions associated with the identified proteins included binding (ion/RNA/enzyme) and maintaining structural integrity. Our results revealed that most of the proteins involved in different pathways (Table S2). Interestingly, we observed that HSPA2 regulates ovary development by changing protein processing in endoplasmic reticulum, and actin plays an important role by regulation of actin cytoskeleton and hippo signaling pathway (Table S2).

## 4. Discussion

Female fertility is largely affected by the quality and health of the ovaries. Effective reproduction is highly dependent on both oocyte quality [12] and follicular development [13]. Normal follicle development is maintained by precise mechanisms which control the primordial follicle formation and the primary follicle activation. In effect, dysregulation of these processes can induce ovarian metabolic modifications that play a pivotal role in aggravating fertility disorders [14]. It is obvious that improper regulation of primordial follicle formation and differentiation could lead to escalated cell apoptosis which can cause premature ovarian failure. Preventing fertility disorders is of great importance; however, little is known about the molecules that regulate normal primordial follicle formation and differentiation. Proteomics approaches have been widely used in agricultural research, in order to investigate the association of specific biological processes with particular molecular functions. In this study, we have applied a 2D-DIGE proteomic analysis approach to identify proteins with distinct molecular functions which can provide a better insight into the underlying molecular mechanism(s) involved in the primordial follicle development. Importantly, some of these proteins have the potential to be used as biological markers for identifying distinct follicular developmental stages in pigs.

### 4.1. Regulation of Primordial Follicle Formation

In mammals, the formation of the primordial follicle is a complex process, involving the breakdown of germ cell cysts, in order to separate the oocytes from each other and subsequently become surrounded by somatic cells. To promote transition of the germ cell cysts into primordial follicles, regulation by cell differentiation factors is required. Actin, which can be found in all eukaryotic organisms, has a key role in ovary differentiation. Actin belongs to a highly conserved family of proteins and is involved in cell shape maintenance, movement, and polarity definition, through regulation of the microtubule cytoskeleton [15]. Defects in actin can disrupt microtubule organization during oogenesis resulting in abnormal cell shape and embryo quality [16, 17], which may induce cell apoptosis. Previous studies in the hamster [16] and rat [17] indicated that distribution of actin is affected by the composition of the culture medium or by organelles, such as mitochondria, which can directly or indirectly affect cell differentiation, leading to failure of the embryos to develop. Notably, actin is a component of primordial oocytes [18] and is involved in the primordial follicle formation by facilitating spindle fiber migration [19–21]. Moreover, actin is crucial for cell differentiation because it forms a scaffold for proteins to generate the physical forces necessary for this process [21]. Our results showed that actin was more abundant at the g55 than the g90 developmental stage and actin involved in regulation of actin cytoskeleton and hippo signaling pathway. The conserved functions of hippo signaling pathway in control of tissue and organ size, cell adhesion junction, and tight junction suggest that actin may regulate follicle development. Interestingly, this coincides with the initiation of the primordial follicle formation at g55, as revealed by our histological observations, indicating that actin may play an important role in this process. Our proteomic analysis has also revealed that gelsolin is lower at the g55 compared to the g90 time-point. Gelsolin is an actin-binding protein and modulation of the actin network has been shown to be involved in cell growth and motility [22]. Interestingly, gelsolin can negatively regulate the expression of apoptosis-associated genes and its overexpression suppresses apoptosis while its downregulation promotes apoptosis [23]. In order for primordial follicle formation to occur, the germ cell cyst must break down and the oogonium be enveloped in a single-cell layer membrane. Unsuccessful completion of this process can lead to upregulation of apoptosis which is required to eliminate malfunctioned oogonia and to protect cell survival; this can further promote activation of primordial follicles to primary follicles. Therefore, lower content of gelsolin and high levels of actin in the ovarian tissues can induce the formation of primordial follicles at the g55 stage of the porcine fetal development.

GO analysis revealed that most of the differentially abundant proteins were involved in anatomical structure development and cytoskeleton organization. RCN3 and RCN1 levels were higher at the g55 than at the g90 stage. RCN3 is a chaperone protein involved in the secretory pathway of the endoplasmic reticulum [24]. This protein is a novel member of the CREC family, which includes RCN1, RCN2, RCN3, SDF4, and CALU proteins [25], and is characterized by its low Ca^2+^-binding affinity. Up to date, there is only one published work concerning the cellular function of RCN3, which has reported that it acts as a chaperone and is involved in the synthesis and secretion of the paired basic amino acid cleaving enzyme 4 [26]. Medical research has revealed that RCN3 is essential for the functional maturation of the lung through regulation of cell proliferation and differentiation [24]. Although not much is known about the molecular function of RCN3, our study has indicated its implication in the primordial follicles formation process. Further investigation of the functional role of RCN3 in the ovary is required in the future. Another member of the CREC family that was identified by our proteomic analysis was RCN1; however there is limited information available about the function of this protein. Only one pervious study has reported that loss of the RCN1 gene renders cells unable to survive [27]. Interestingly, it has been shown that the levels of the RCN1 protein are higher during the primordial follicles formation (embryo development) stage in mice [28], which is in accordance with our pig results.

Overall, our results revealed that actin, gelsolin, RCN3, and RCN1 are associated with primordial follicle formation and although the mechanisms of action of these proteins are still unknown, they might play an important regulatory role in this process.

### 4.2. Regulation of Primordial Follicle Differentiation into Primary Follicle

After primordial follicles are formed, some begin to be activated and are selected for further development while the remaining ones become quiescent until the next cycle of activation [29]. Activated primordial follicles will first transition into primary follicles which are characterized by the presence of a single layer of cuboidal follicular cells surrounding an oocyte [30]. Next, the primary follicles increase in size, the granulosa cells differentiate to form multiple layers, the oocytes grow, and the somatic cells accumulate more layers [31]. Therefore, in order for the primordial follicle transition into primary follicles to occur, regulation by cell proliferation factors is required. The major vault protein (MVP) is a vital component and marker of the vault [32], which plays an important role in follicle activation [33] probably through inhibition of the PTEN and the estrogen receptors [34, 35]. In the present study, histological examination revealed that the primordial follicles transition to primary follicle begins at g90 which corresponds to the time-point when the levels of the MVP protein are increased, relative to the g55 stage. One possible explanation is that the high content of MVP directly inhibits PTEN signaling pathways and consequently promotes follicular growth and regulates follicular activation [36–38]. This could have enabled optimal activation of primordial follicles and prevented the reduction of the follicle pool (prematurely activated primordial follicles), hence rendering MVP a key player for the follicle differentiation. Moreover, these results revealed that MVP might have the potential to be used as a protein marker specifically for the induction of ovarian development, which will be investigated in the future.

### 4.3. Regulation of Stress Response

Primordial follicle formation and primordial follicle transition to primary follicle stages are characterized by high protein turnover which requires the upregulation of protein synthesis and catabolism. This can lead to accumulation of unfolded proteins in the cells that can trigger stress response mechanisms. HNRNPK belongs to the family of heterogeneous ribonucleoproteins (hnRNPs) and is involved in the mRNA processing and export [39], chromatin remodeling [40], and transport [41]. The HNRNPK can protect its target mRNAs through maintenance of cellular ATP levels under stress conditions [42]. Fetal ovaries constantly receive nutrients from mothers via the placenta; therefore, upregulation of HNRNPK at g55 may have an important function in stabilizing fetal cell homeostasis during ovarian growth. Interestingly, this hypothesis is supported by rat studies [43] which have shown that reducing the HNRNPK mRNA levels of neonatal ovaries of rats resulted in a substantial loss of naked oocytes and primordial follicles, indicating that HNRNPK is a key regulator for primordial follicle formation.

Another stress response protein that was identified in our study was the heat shock-related 70 kDa protein 2 (HSPA2), whose content was higher at g90 than at g55 time-point. The HSPA2 belongs to the family of HSP, which act as chaperones and involved in crucial cellular functions in all eukaryotic organisms [44]. HSPA2 is involved in cancer cell survival in many tissues [45] and is essential for spermatogenesis [46]. Although HSPA2 is highly specific to male fertility, recent studies have shown that HSPA2 is expressed in various types of somatic tissues, for example, in the brain, pituitary, spleen, ovary, oviduct, and the uterus [47]. Interestingly, Wisniewska et al. (2010) found that HSPA2 can bind and hydrolyze ATP, whose products retain bound during crystallization [48]. In addition, when the cytoplasmic ATP concentration is increased, nucleotide dissociation becomes a rate-limiting step for substrate release [49]. The transition of primordial follicle to primary follicle is a high energy demanding process which requires consumption of ATP and probably leads to reduction of ATP levels. In our study, this would inhibit binding of substrate to HSPA2 and consequently release the HSPA2, resulting in increased levels of this protein at the g90 stage of fetal ovary development. Another reason for the increased HSPA2 levels could be due to increased protein synthesis during the process of transition, and this coincides with our result that the HSPA2 involved in protein processing in endoplasmic reticulum. This could lead to high levels of unfolded proteins resulting in increased cellular stress. In order for the cell to relieve this stress it possibly upregulates the expression of the stress response genes, resulting in the increased levels of chaperones.

Collectively, our results revealed that HNRNPK may be involved and play an important role in primordial follicles formation, while HSPA2 may potentially be used as a suitable biomarker for the transition of primordial follicles to primary follicles. The mechanism(s) by which these stress response proteins interact with ovarian development remains to be elucidated.

## 5. Conclusions

In the present study, we applied a 2D-DIGE based proteomic analysis to investigate the molecular mechanism for the primordial follicle formation and differentiation in pigs. Our results suggest that the high level of actin, RCN1, RCN3, and HNRPK at the g55 developmental stage may be related to primordial follicle formation in pigs, whereas the high levels of MVP and HSPA2 at g90 indicate that they might be associated with the regulation of primordial follicle transition to primary follicle. These findings contribute to a better understanding of porcine fetal ovary development.

## Supplementary Material

Table S1: Number of proteins annotations. All identified proteins were functionally annotated in Go database according to their molecular, biological process, and cellular component.

## Figures and Tables

**Table 1:** Differentially expressed proteins in the ovary on days 55 and 90 of gestation.

Number	Protein IDs	Mascot score	Mr	PI	Fold change	Coverage	Protein name
Anatomical structure development							

S30	F1RS36	478	70323	5.21	−2.94988	33%	78 kDa glucose-regulated protein
S1	F1SDX6	488	78202	5.19	+1.60756	28%	High similarity protein-glutamine gamma-glutamyltransferase 2
S15	F1SGP8	169	38756	4.74	+4.17177	24%	High similarity reticulocalbin-1
S18	I3LMU6	231	37602	4.88	+5.07398	32%	High similarity reticulocalbin-3
S16	I3LMU6	194	37602	4.88	+10.8714	19%	High similarity reticulocalbin-3
S32	F1RKM0	346	66674	5.08	−2.016	38%	High similarity lamin-B1

Cytoskeleton organization							

S22	TBB5	194	50095	4.78	+3.63517	16%	Tubulin beta chain
S28	GELS	118	85065	5.93	−2.19131	8%	Gelsolin
S39	TPM4	314	28619	4.67	−1.65209	39%	Tropomyosin alpha-4 chain

Response to stress							

S19	F1SRK6	282	92633	5.03	+10.0021	13%	Endoplasmin
S29	F1RQU2	120	83543	4.96	−2.51883	18%	High similarity heat shock protein HSP 90-beta
S6	F1SMZ7	162	61266	5.61	+1.83048	20%	High similarity 60 kDa heat shock protein, mitochondrial
S10	I3LQS0	89	49512	5.38	+5.17595	14%	High similarity heterogeneous nuclear ribonucleoprotein K
S5	I3LNG8	327	48248	7.01	+1.61547	44%	High similarity stress-induced phosphoprotein 1
S31	F1SA70	329	70065	5.51	−4.51308	25%	High similarity heat shock-related 70 kDa protein 2

Cell differentiation							

S36	C7AI81	367	42381	5.23	−1.63319	36%	Actin alpha 2
S27	F1S596	188	61481	4.36	−1.64581	11%	High similarity glucosidase 2 subunit beta
S20	I3LVD5	377	42108	5.31	+5.52608	34%	Actin, cytoplasmic 1
S21	I3LVD5	294	42108	5.31	+1.7995	33%	Actin, cytoplasmic 1
S23	VIME	408	53692	5.06	+2.45187	36%	Vimentin

Biosynthetic process							

S35	B26/I3LK59	240	38173	8.93	−2.02688	16%	Enolase
S37	F1SQ06	150	21243	4.83	−3.32526	31%	High similarity spermine synthase

Cell proliferation							

S25	I3LQ79	477	99973	5.53	−3.23725	23%	High similarity major vault protein
S26	I3LQ79	566	99973	5.53	−25.8052	32%	High similarity major vault protein
S40	APOA1	177	30307	5.48	−1.62505	40%	Apolipoprotein A-I

Transport							

S3	FETA	390	70405	5.47	+2.00804	30%	Alpha-fetoprotein
S4	A2THZ2	325	71550	5.92	+32.0392	29%	Albumin
S42	A2THZ2	488	71550	5.92	−2.71787	35%	Albumin

Protein folding and maturation							

S2	F1S0M9	174	34453	8.24	+6.61563	33%	Peptidyl-prolyl cis-trans isomerase
S34	F1RP17	246	61756	6.79	−2.44313	19%	T-complex protein 1 subunit gamma

Small molecule metabolic process							

S13	Q2HPK3	381	33321	5.86	+1.86412	35%	Tubulin alpha-3 chain
S41	A6M930	193	46601	5.33	+1.75218	32%	Eukaryotic translation initiation factor 4A isoform 2

Others							

S7	A1AT	125	47449	5.54	+2.38373	9%	Alpha-1-antitrypsin
S8	F1RIP6	188	53170	5.02	+2.21427	30%	High similarity nucleobindin-1
S9	F1SCD0	103	46782	5.8	+2.93238	17%	Uncharacterized protein
S11	F1S1U5	164	47140	4.7	+11.3392	17%	High similarity Golgi reassembly-stacking protein 2
S12	F1SME6	331	56996	6	+3.44637	28%	Uncharacterized protein
S14	F1SFI6	310	43561	7.87	+3.28303	32%	Uncharacterized protein
S17	KCRB	129	11670	5.19	+2.12203	28%	Creatine kinase B-type
S24	Q1T7A9	82	10639	5	−1.76143	27%	Type VI collagen alpha-1 chain
S33	I3L954	184	60941	5.55	−1.78172	14%	Uncharacterized protein
S38	F2Z5C1	229	33193	4.95	−1.94943	36%	Annexin
S43	I3L954	64	60941	5.55	−2.06063	9%	Uncharacterized protein

Mr: relative molecular mass; PI: isoelectric point. Symbols (+) and (−) denote an increase and a decrease, respectively, at day 55 of gestation of fetal ovaries, when compared with day 90 of gestation of fetal ovaries. High similarity indicated that those proteins are from mouse databases (https://www.ncbi.nlm.nih.gov/protein/?term=txid10088[Organism:exp]).
